# Readily Functionalizable and Stabilizable Polymeric Particles with Controlled Size and Morphology by Electrospray

**DOI:** 10.1038/s41598-018-34124-0

**Published:** 2018-10-24

**Authors:** Hoik Lee, Sol An, Sukjoo Kim, Bokyoung Jeon, Myungwoong Kim, Ick Soo Kim

**Affiliations:** 1 Nano Fusion Technology Research Group, Division of Frontier Fibers, Institute for Fiber Engineering (IFES), Interdisciplinary Cluster for Cutting Edge Research (ICCER), Shinshu University, Tokida 3-15-1, Ueda, Nagano, 386-8567 Japan; 2 Department of Chemistry and Chemical Engineering, Inha University, Incheon, 22212 Korea

## Abstract

Electrospraying is an effective and facile technique for the production of micro- or nanoparticles with tailored sizes, shapes, morphologies, and microstructures. We synthesized functionalizable poly(styrene-*random*-glycidyl methacrylate) copolymers and used them to fabricate microparticles via the electrospray technique. The sizes and morphologies of the electrosprayed particles are controlled by altering the process parameters (feed rate and applied voltage), and the composition and thermodynamic properties of the polymer (i.e., compatibility of the polymer with the solvent). We further investigated modifying the surfaces of the electrosprayed particles with 3-mercaptopropionic acid by a simple and efficient thiol-epoxy “click” reaction as a proof-of-concept demonstration that desired functionality can be introduced onto the surfaces of these particles; the outcome was confirmed by various spectroscopic techniques. In addition, the epoxides within the particles easily undergo crosslinking reactions, enabling further effective particle stabilization. The results reveal that the structure and properties of the polymer can be used to fine-tune the structural parameters of the electrosprayed particles, such as their sizes and morphologies, which opens up the possibility of imparting a variety of desired chemical functionalities into the structures of stable organic materials via post-electrospray modification processes.

## Introduction

Interest in organic micro/nanoparticles has rapidly grown over the past few decades due to their applications to areas that include sensing, pharmaceutics, and catalysis, among others^1–3^. Typically, such particles are fabricated using polymeric materials, as the desired properties of a selected polymer can be beneficial for tailoring the functionalities of the particles. For example, poly(ethylene glycol) microparticles can be used in biopharmaceutical applications such as drug delivery systems^4^. In order to efficiently synthesize these particles, a variety of techniques have been developed; examples include suspension/emulsion polymerization^5^, solvent evaporation^6^, microfluidics^7^, and electrospray^8^. Among these methods, suspension/emulsion polymerization techniques are predominantly used due to their accessibilities and scalabilities^9^. In addition, the electrospray technique has attracted much attention as it is a facile and suitable method for the formation of polymeric particles with highly controlled sizes, dispersities, unconventional shapes, and unique surface morphologies that cannot be achieved by other methods^10,11^. Electrospray provides unique approaches for addressing significant challenges; for instance, replacing red blood cells with polymeric materials in bioanalysis technologies resolves issues such as biological hazards, instability, and significant variations in analytical results^3,12^.

Electrospray uses an electrostatic field to form liquid jets from the tip of a capillary connected to a feed source. A polymer solution is injected from the source to the nozzle where the electrical potential is applied. If the applied field is sufficiently intense, charges on the liquid drop formed at the end of the nozzle induce electrical stress that results in the formation of a distorted hemispherical liquid drop of conical shape at the end of the capillary^13,14^. Above a critical voltage, a stable liquid jet is formed, which then disintegrates into a number of droplets that are accelerated onto the collector. If a polymer solution is sprayed, micro/nanoparticles are fabricated through the instant and complete evaporation of the solvent from the solution droplets during their flight to the ground collector. This process is referred to as “electrospray”. This technique facilitates the preparation of a wide range of well-defined polymer films with self-assembled structures, polymeric particles, and nanofibers with a variety of functionalities^15–18^. The simplicity, flexibility, and efficiency of this technique for the generation of particles with desired sizes and shapes have led to electrospray becoming a promising and well-established approach for industrial applications.

More importantly, electrospray can be advantageous for the fabrication of functionalizable particles. The incorporation of desirable functionalities is essential for expanding the applicability of functional polymeric particles. Conventionally, emulsion/suspension polymerization of functional monomers has been employed for the preparation of functional polymer particles. However, this method does not allow the incorporation of highly reactive functional groups that cannot survive during polymerization. To circumvent this issue, the desired functionality can be introduced through the chemical modification of the fabricated particles; however, introducible functionalities are limited by the chemistry used during modification that often requires harsh chemicals. Modifications within these particles are also limited; only the surfaces are modifiable, leaving the insides of the particles intact. In addition, the resulting objects typically exhibit spherical morphologies; consequently, the final shapes and surface morphologies cannot be further tailored.

**Figure 1:**
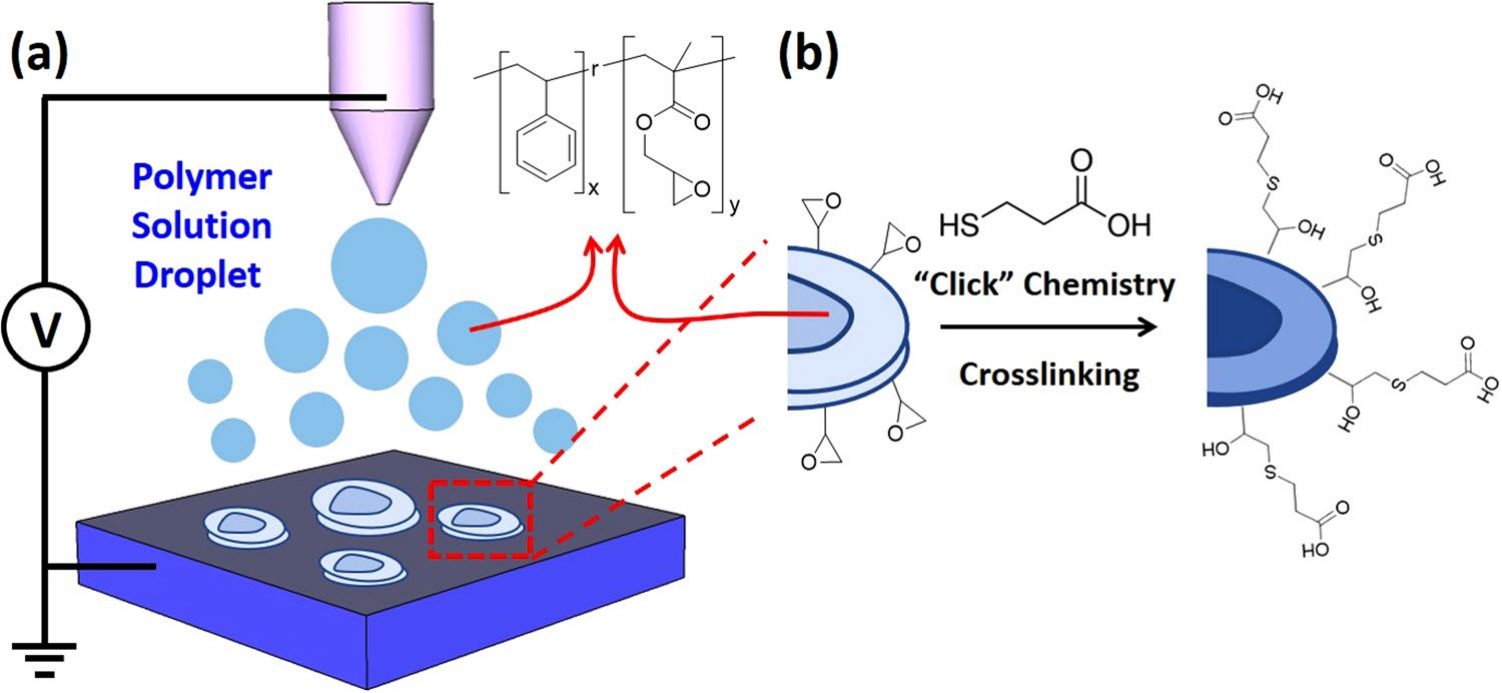
Schematic representation of (**a**) the fabrication of P(S-*r*-GMA) particles through electrospraying, (**b**) functionalization of an electrosprayed-particle surface with MPA via thiol-epoxy click chemistry and concurrent stabilization through crosslinking.

To address these challenges, we fabricated functionalizable microparticles with controlled sizes and morphologies by electrospray. The epoxide was chosen as a model reactive group. Epoxides were incorporated into the polymer chain through the synthesis of copolymers consisting of glycidyl methacrylate and a comonomer (Fig. 1). The glycidyl group is very reactive toward many functional groups; consequently a number of functionalities can be subsequently introduced into the polymer chain^15,19^. Furthermore, under specific conditions, the polymer sample can undergo crosslinking reactions that help to stabilize the particles against exposure to chemicals and other stimuli^20^. For particle morphology, the solidification of the electrosprayed droplet is known to be affected by many parameters, including volatility, viscosity, surface tension, and solution concentration, as well as the structural parameters of the polymer that affect its interactions with solvent molecules and the final morphology^10,21^. In addition, since microparticles consisting of polymers with the limited range of structure such as homopolymers and block copolymers have been mainly explored^22,23^, the effect of polymer structure is not fully revealed yet.

In order to fully understand the process for the fabrication of reactive particles with controlled sizes and shapes, for the proof of concept we first designed and synthesized a series of poly(styrene-*random*-glycidyl methacrylate) (P(S-*r*-GMA)) copolymers in which both composition and molecular weight were systematically varied. This simple type of copolymer exhibits a wide range of surface-engineering applications; for example, it can be used to fabricate crosslinked thin films that are able to have their surface activities and energies toward a wide range of substrates precisely tuned by controlling the copolymer composition^24–26^. In addition, their hydrophobic properties, due to the presence of styrene, facilitate post-electrospray modification with water-soluble molecules. With these copolymers we investigated the effect of polymer compatibility with the solvent, which is strongly related to the copolymer composition, the solvent, and the electrospray process parameters, namely the solution feed rate and the voltage. The results reveal that the sizes, shapes, surface smoothness, and porosities of the P(S-*r*-GMA) particles are governed by the parameters mentioned above. Fabricated particles were further subjected to post-electrospray modification via the simple and rapid thiol-epoxy click reaction that proceeds quantitatively. The epoxides on the surfaces of the resulting particles were converted into carboxylic acids through their reactions with 3-mercaptopropionic acid (MPA). In addition, during these functionalization reactions, the epoxy groups inside the particles underwent crosslinking, which provides a route that concurrently achieves both successful particle functionalization and stabilization. Our findings further highlight the use of the electrospray technique for enabling access to new functionalizable organic materials platforms that are widely able to be post-electrospray functionalized. This process represents an efficient route to versatile functional surfaces for desired applications.

## Results and Discussion

### Random copolymerization of styrene and GMA

P(S-*r*-GMA) was synthesized by conventional free-radical polymerization, which is a simple, easily accessible, and scalable polymerization method^24^. We aimed to vary the composition of the random copolymer and its molecular weight in order to systematically control the number of functionalizable epoxides and to investigate the effect of molecular weight (M_n_) and polar-monomer incorporation on the morphologies of the electrosprayed particles. At *f*_st_ = 0.600, M_n_ was controlled in the 14.8–125.6 kg/mol range by varying the amount of initiator ([M]_0_:[I]_0_ = 76–668) (Table 1). The actual styrene compositions of the copolymers (*F*_st_) were determined by ^1^H-NMR analyses and were well-controlled in the 0.550–0.580 range in all samples. When *f*_st_ = 0.800, a sample with a medium M_n_ (~63.4 kg/mol) and a reasonable *F*_st_ (0.721) was successfully synthesized at [M]_0_:[I]_0_ = 593 (other reaction conditions were identical to those used when *f*_st_ = 0.600). However, polymerization at *f*_st_ = 0.200 was problematic using the conditions described above. For example, polymerizations for 22, 6, and 4 h resulted in insoluble solid chunks that suggest crosslinking during polymerization. Therefore, the polymerization time was reduced to 2.5 h, which afforded a polymer sample with an M_n_ of 76.4 kg/mol, narrow dispersity, and a reasonable *F*_st_. Well-defined random copolymer samples with systematic variations in composition and molecular weight were obtained through careful optimization; these samples were used in further studies to fabricate reactive particles.

**Table 1:** Properties of a series of synthesized P(S-*r*-GMA) copolymers with various molecular weights and compositions.

Sample	[M]_0_:[I]_0_	Reaction time (h)	M_n_^a^ (kg/mol)	*Ð* ^*a*^	*f*_st_	*F*_st_^b^
SG0.8-63 K	593	22	63.4	1.69	0.800	0.721
SG0.6-15 K	76	22	14.8	1.85	0.600	0.579
SG0.6-24 K	151	22	24.2	2.19	0.600	0.563
SG0.6-53 K	302	22	52.7	3.29	0.600	0.575
SG0.6-60 K	422	22	59.6	2.23	0.600	0.552
SG0.6-126 K	202	22	125.6	2.52	0.600	0.546
SG0.2-174 K	668	2	173.7	2.29	0.200	0.260
SG0.2-76 K	419	2.5	76.4	2.13	0.200	0.241

^a^Molecular weights and dispersities were determined by SEC. ^b^Compositions were determined using quantitative ^1^H-NMR analyses.

### Electrospraying the synthesized reactive copolymers: concentration and molecular weight dependences

**Figure 2:**
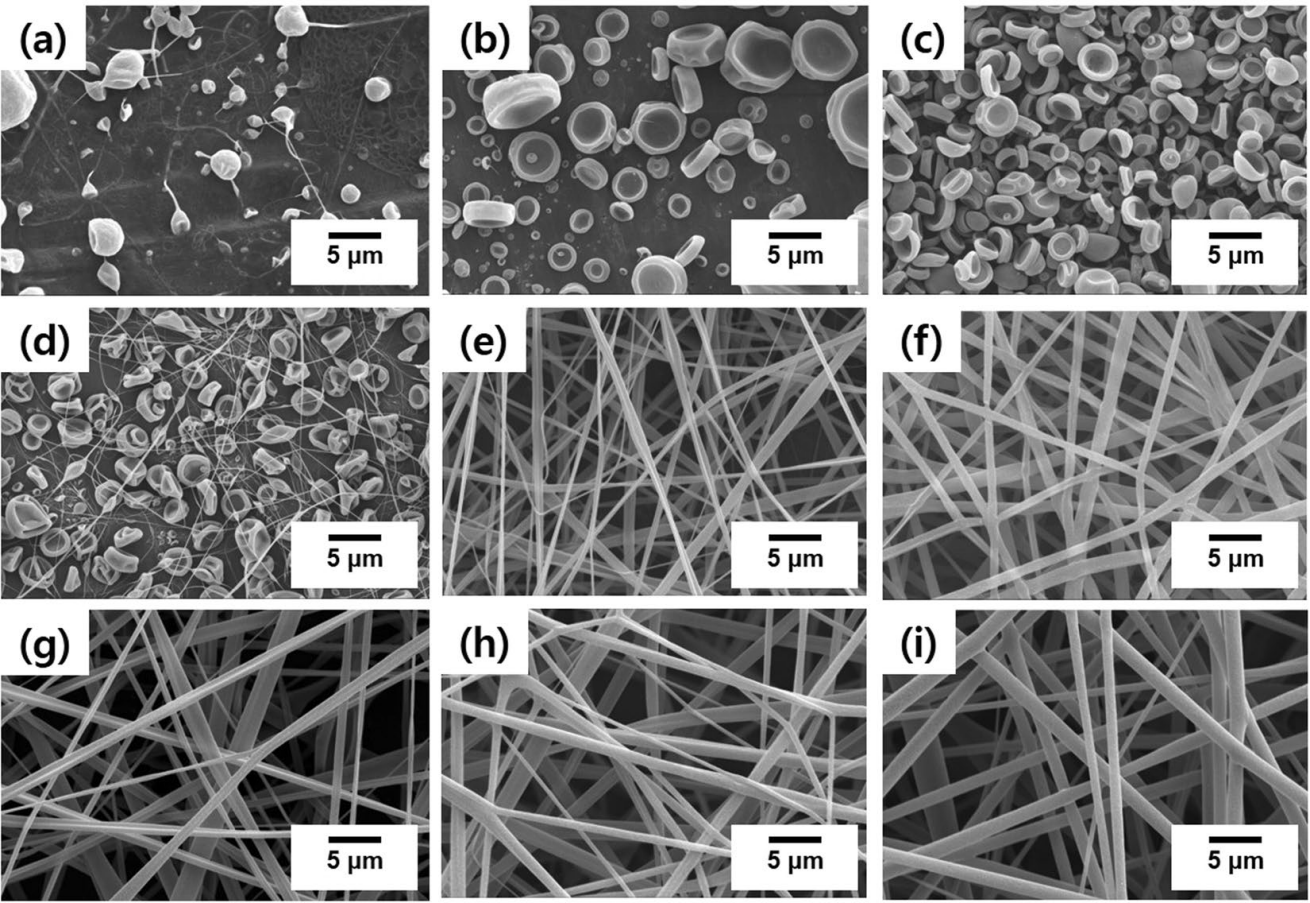
SEM images of electrosprayed/electrospun particles/nanofibers with varying polymer solution concentrations: (**a**) 1 wt%, (**b**) 3 wt%, (**c**) 5 wt%, (**d**) 10 wt%, (**e**) 14 wt%, (**f**) 15 wt%, (**g**) 16 wt%, (**h**) 17 wt%, and (**i**) 18 wt%.

When electrospraying a polymer solution, the resulting morphology is highly dependent on the viscoelasticity and the surface tension of the solution, which is controlled by its concentration; therefore, the concentration is typically the first parameter varied when examining the morphologies that are achievable by the system. The polymer solution is ejected through the jet over a few milliseconds in the presence of an electric field; consequently, numerous polymer solution drops are formed. If the concentration is too low, the solution does not exhibit sufficiently high surface tension; hence, the viscoelastic force required to stretch the solution droplet in order to form a nanofibrous structure cannot be applied during electrospray. Furthermore, the deposition is performed at room temperature far below glass transition temperature (T_g_) of the series of copolymers expected to fall in the range between the T_g_ of PS (~373 K) and the T_g_ of PGMA (~336 K), which is a necessary condition for the electrospray deposition^27^. As a result, the polymer solidifies during solvent evaporation, leading to the formation of micro/nanoparticles. Figure 2 displays SEM images of electrosprayed polymeric particles and nanofibers fabricated from MEK solutions of P(S-*r*-GMA) (SG0.6-60 K) at concentrations in the 1–18 wt% range. At low concentrations (3–5 wt%) microsized particles exhibiting nonspherical jellyfish-like shapes were fabricated (Fig. 2b,c); in particular, particles prepared from the 5 wt% solution were observed to be more uniform and to be in greater quantities than particles made at lower concentrations. The transition from particles to nanofibers was clearly observed with further increases in concentration, to 10 wt%, indicating that sufficient viscoelasticity was achieved in order to stretch the droplets and form fibrous structures (Fig. 2d). Nanofibers were successfully fabricated at concentrations above 10 wt%; the fiber diameter increased from 0.44 ± 0.12 to 1.23 ± 0.22 µm as the concentration increased from 14 to 18 wt%, which are typical changes in diameter observed in electrospinning experiments^28^ (Fig. 2e–i).

**Figure 3:**
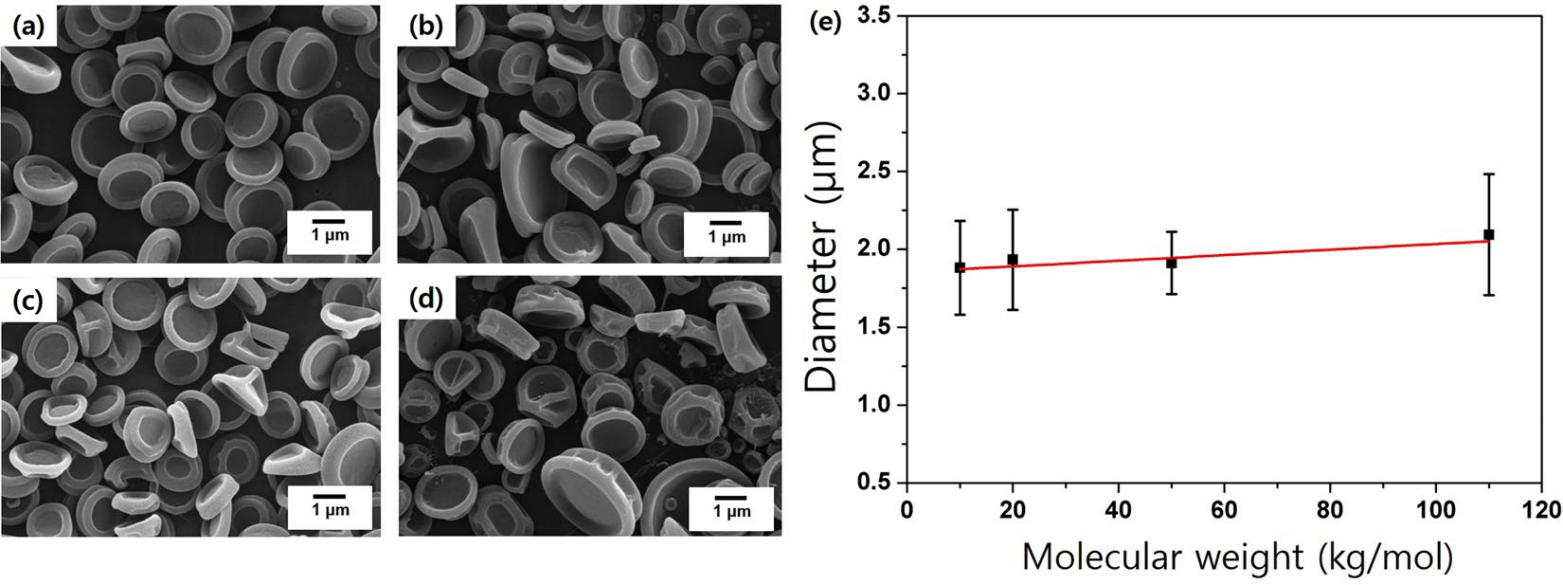
SEM images of electrosprayed particles of varying molecular weight: (**a**) 14.8 kg/mol, (**b**) 24.2 kg/mol, (**c**) 52.7 kg/mol, and (**d**) 125.6 kg/mol. (**e**) Particle size as a function of molecular weight. The red line represents a linear fitting result.

We further investigated the effect of the molecular weight of the copolymer on the final morphology. Since we were interested in particles, the solution concentration was fixed at 5 wt%, which was optimum for the formation of well-defined particles. The process parameters used were 15 cm, 11 kV, and 0.6 mL/h for the tip-to-collector distance, voltage, and feed rate, respectively. Figure 3 displays SEM images of P(S-*r*-GMA) microparticles (*f*_st_ = 0.600) formed with M_n_ values in the 14.8–125.6 kg/mol range. No significant change was observed in the nonspherical shapes of these particles, although the average particle diameter and its standard deviation were slightly higher in the high molecular weight sample (SG0.6-126 K), which is attributed to the increase in the viscosity of the polymer solution due to the higher molecular weight. These results reveal that molecular weight does not considerably affect the morphologies of the microparticles formed by electrospraying; therefore, random copolymers in the 60–75 kg/mol molecular weight range were further investigated systematically in order to discover other relevant parameters that govern the morphologies of these microparticles.

### Effective particle size control through adjustment of process parameters: flow rate and electrospray voltage

**Figure 4:**
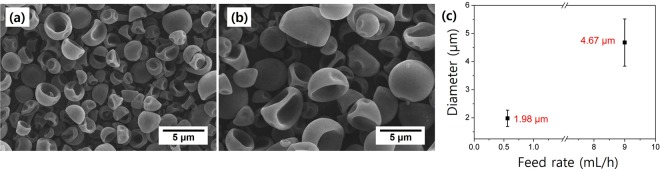
SEM images of electrosprayed particles formed at different feed rates: (**a**) 0.6 mL/h, and (**b**) 9 mL/h. (**c**) Particle size as a function of flow rate.

The electrospray process parameters can be adjusted to effectively control the sizes and shapes of the particles in order to achieve desirable morphologies. Specifically, it is known that applied voltage and flow rate are crucial parameters that significantly affect the sizes of the resulting particles^29^. The flow rate should first be optimized in a range suitable for successful electrospraying; if the flow rate is too slow, rapid evaporation of a volatile solvent prior to the formation of a jet can lead to blockage of the ejection needle. On the contrary, if the flow rate is too fast, droplets cannot form and the solution is directly ejected onto the surface. More importantly, flow rate control is an easy and powerful method for obtaining particles of the desired target size. The sizes of the electrosprayed droplets and resulting particles are highly correlated with flow rate: the diameter systematically increases when the flow rate becomes high^30^. As expected, flow rate variation was highly effective in our system; electrospraying a P(S-*r*-GMA) solution at low (0.6 mL/h) and high (9 mL/h) flow rates resulted in average particle sizes of 1.98 ± 0.29 and 4.67 ± 0.64 µm, respectively, without any significant change in particle shape (Fig. 4). We note that electrospraying at high flow rates resulted in the formation of satellite droplets that led to much larger particles than average.

**Figure 5:**
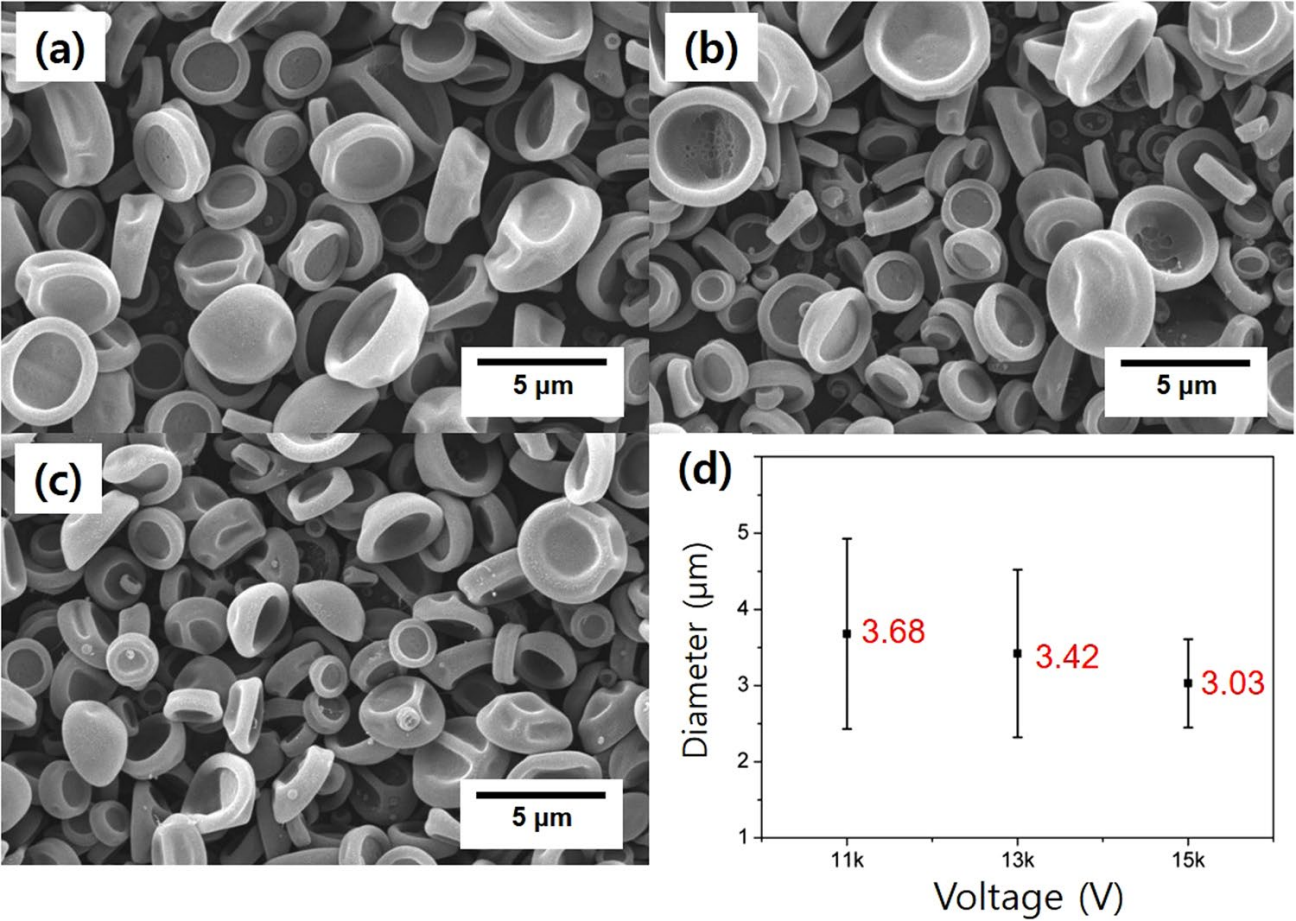
SEM images of electrosprayed particles prepared at different voltages: (**a**) 11 kV, (**b**) 13 kV, and (**c**) 15 kV. (**d**) Particle size as a function of applied voltage.

The applied voltage is equally important to obtain the target particle morphology, as the driving force for particle formation is provided by the electrical potential. Sufficient electrical force is required to overcome the surface tension of the sprayed solution in order to successfully generate a Taylor cone and the subsequent formation and acceleration of droplets. Cloupeau *et al*. reported that the electrospray process can be sustained in a variety of modes at different voltages^31^. These modes are characterized by the shapes of the surfaces from which the liquid jet originates, and significantly affect droplet size and size distribution. In our studies, P(S-*r*-GMA) particles were also observed to be affected by the applied voltage. The typical range of applied voltages used for reliable electrospraying has been established to be 10 to 15 kV; therefore, we studied the effect of voltage variation in this range. As shown in Fig. 5, the sizes of the electrosprayed particles were effectively controlled through voltage variation. The process parameters used were 15 cm, and 0.9 mL/h for the tip-to-collector distance, and flow rate, respectively. Decreases in the average size and its standard deviation were observed with increasing voltage; the average particle sizes were 3.68 ± 1.24 μm, 3.42 ± 1.10 μm, and 3.03 ± 0.58 μm at 11, 13, and 15 kV, respectively. On the other hand, all of the electrosprayed particles exhibit similar jellyfish-like nonspherically structured morphologies, indicating that morphology is not critically affected by voltage. These studies reveal the sizes of the reactive P(S-*r*-GMA) particles are readily controlled using typical electrospray methods.

### Morphological changes induced by polymer solution behavior: solvent volatility and quality

During electrospray, the solvent plays a critical role that affects many aspects, as its physical properties such as conductivity, surface tension, viscosity, and vapor pressure have a significant impact on the particle formation process. In particular, polymer solidification from an electrosprayed droplet is governed by two main parameters: solvent evaporation and polymer diffusion during the flight of the solution droplet to the collector, which also strongly affect the final morphology of the electrosprayed particle^32,33^. If the solvent molecules in a droplet evaporate rapidly during flight due to their high volatility, the droplet size will change rapidly. An increase in the rate of change in the droplet radius *r* (shrinking velocity, *R*_r_ = ∂*r*/∂*t*) will lead to a more kinetically trapped morphology as the system has less time for its polymer chains to arrange themselves compared to a droplet composed of a less volatile solvent. In this case, the polymer chain arrangement behavior to form solid particles is described by the diffusion coefficient (*D*) of the chains in the flying droplet. Correlations between particle morphology and two physical parameters were described through the introduction of a modified Peclet number (*P*_*e*_)^34^, and is given by the equation: *P*_*e*_ = *R*_r_·*r*/*D*, which emphasizes that the shape of a particle is governed by the competition between solvent-molecule evaporation and polymer diffusion in the droplet. If *P*_e_ is very high (highly volatile solvent or slow polymer diffusion), then the solvent evaporates from the surface of the droplet before polymer chains can arrange themselves and rehomogenize, resulting in high polymer concentrations near the surface. Further solvent evaporation from the surface leads to the formation of a thin solid crust covering a hollow sphere, which subsequently collapses to a particle that exhibits biconcave morphology similar to that of a human red blood cell. When *P*_e_ is low (less volatile solvent or fast polymer diffusion), the polymer chains have sufficient time to homogenize during the shrinkage of the droplet, resulting in a less hollow and spherical particle with a smooth surface. We note that determining precise values of *R*_r_ and *D* in droplets is complicated, and this has limited the ability to fully interpret a variety of systems. In spite of this, the Peclet number has been beneficial for systematically describing and predicting the results of electrospray processes.

**Figure 6:**
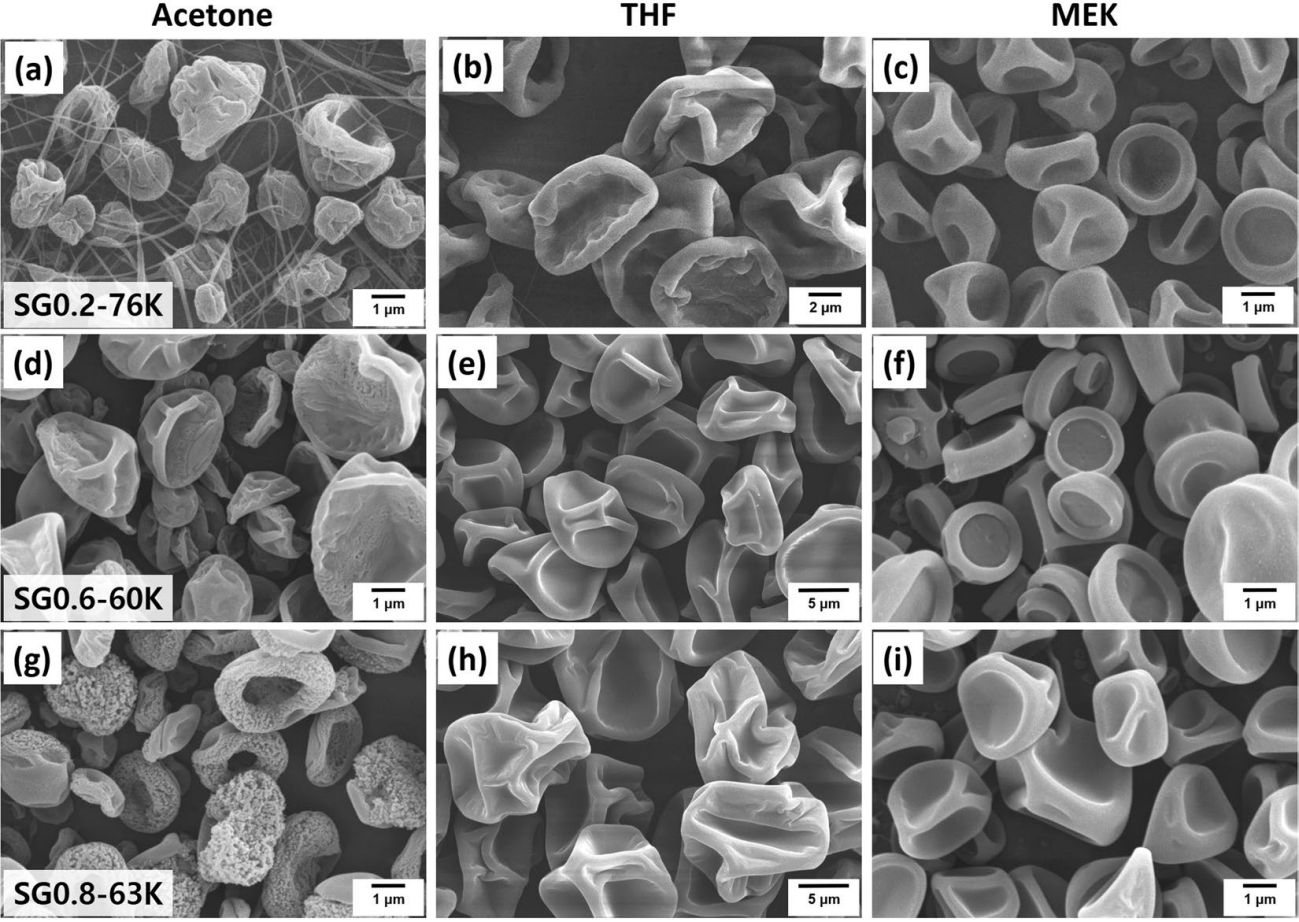
SEM images showing the morphologies of electrosprayed particles as functions of solvent and copolymer composition: (**a**–**c**) SG0.2-76 K in acetone, THF, and MEK, respectively; (**d–f**) SG0.6-60 K in acetone, THF, and MEK, respectively; and (**g**–**i**) SG0.8-63 K in acetone, THF, and MEK, respectively.

Experimentally, volatility can be controlled by changing the solvent. In order to systematically study control over the final morphology, we carefully selected three solvents capable of dissolving a series of synthesized P(S-*r*-GMA) copolymers, which also have systematic variations in volatility: acetone (b.p. = 56 °C, *P*_vap_(25 °C) = 229.5 torr), THF (b.p. = 66 °C, *P*_vap_(25 °C) = 162.2 torr), and MEK (b.p. = 79 °C, *P*_vap_(25 °C) = 100.0 torr). Figure 6d–f show SEM images of microparticles fabricated by electrospraying SG0.6-60 K solutions prepared using the three above-mentioned solvents. All particle morphologies were observed to be somewhat biconcave and corrugated in shape, suggesting that the Peclet numbers of the corresponding droplets are all similar, a result of the competition between *R*_r_ and *D*, as described above. However, it should be noted that the surface roughnesses of the particles formed using the various solvents were observed to be very different. Particles fabricated using acetone exhibited rough and somewhat porous surfaces compared to the smooth surfaces of the particles fabricated using THF or MEK. This trend was also observed for SG0.8-63 K; more importantly, particles fabricated from acetone exhibited even rougher and more porous surface morphologies than those observed for SG0.6-60, strongly suggesting that the composition of the copolymer has an impact on the final morphology.

The variable that we created in our system is the copolymer composition, which affects the interactions of the polymer chains with the surrounding solvent molecules; i.e., solvent quality. Li *et al*. demonstrated a nonsolvent-assisted electrospraying method for the fabrication of porous poly(methyl methacrylate) (PMMA) microspheres^35^. Small amounts of a nonsolvent for PMMA (hexanol) was added to a dichloromethane/PMMA solution under controlled humidity condition to promote nonsolvent-induced phase separation in the electrosprayed droplets, which affords porous structures after solvent evaporation. This method has been expanded to another type of homopolymer, e.g. poly(ε-caprolactone), to effectively fabricate wide range of microstructure including spheres and fibers with controlled surface morphology^36^. These studies indicate that solvent quality significantly affects the polymer chains that control the surface morphology. Solvent quality can be evaluated using the Hildebrand solubility parameters, which enable us to estimate interactions in the polymer solutions in a simple manner; these parameters for PS, PGMA, acetone, THF and MEK are 18.5, 19.4, 19.9, 19.4 and 19.3, respectively^37^. MEK and THF are good solvents for PS and PGMA, since their solubility parameters are comparable to those of PGMA and PS. However, since acetone is well known to be a poor solvent for PS, polymer chains with higher *F*_st_ values tend to prefer a more collapsed state than those with lower *F*_st_ values.

When homopolymers were electrosprayed, very smooth and slightly collapsed spherical structures were observed for PS, and more collapsed and biconcave spherical structures were observed for PGMA (Fig. S1). We note that the PS solution in acetone could not be electrosprayed due to its poor solubility. This implies that the PS polymer chains have little chance of arranging and diffusing during solvent evaporation during the electrospray process; consequently, porous structures are obtained, as shown in Fig. 6g. Particles fabricated using SG0.6-60 K in acetone exhibited corrugated rather than porous surfaces (Fig. 6d), indicating that the amount of styrene in the polymer chain is insufficient to make the solvent poor enough for pore formation. Interestingly, the lower *F*_st_ copolymer, SG0.2-76 K, exhibits concurrent formation of both microparticles and nanofibers, as shown in Fig. 6a, indicating that the ejected droplets from the jet became stretched to the counter-electrode to form nanofibers. This is ascribable to the large amount of PS present in P(S-*r*-GMA), which collapses the polymer and possibly induces higher local concentrations of GMA in the solution droplets. As a result, the highly concentrated PGMA portion in the droplet stretches during flight leading to nanofibers. Therefore, understanding and controlling the interactions between the polymer chains and the solvent molecules enable rational access to controlled surface morphologies, from nanoporous structures less than 50 nm in size, to smooth structures on the particles.

### Post-electrospray modification and stabilization of fabricated particles

Having successfully fabricated reactive polymeric particles, we further studied the post-electrospray modification of these particles using simple and efficient functionalization chemistry, as these copolymers are designed to allow the introduction of new functionalities through their existing glycidyl groups. The ring strain in an epoxide makes this functional group susceptible to nucleophilic attack that opens the strained three membered ring, thereby recovering the ideal tetrahedral carbon angle. This reactivity can be exploited through the use of a thiol and an alkaline catalyst; the thiol-epoxy “click” reaction is classified as a rapid click reaction since it usually affords products in quantitative yields. The hydroxyl group formed during the click reaction provides versatility as it can be further functionalization; hence a variety of dual functional materials can be obtained in this manner^38^. We selected MPA as a model proof-of-concept reactant with which to functionalize the fabricated particles, as it is soluble in water allowing the fabricated particles to preserve their morphologies during functionalization. In addition, the versatility of the carboxylic acid facilitates dispersibility in water-based media and numerous chemical routes for further complex surface functionalization.

**Figure 7:**
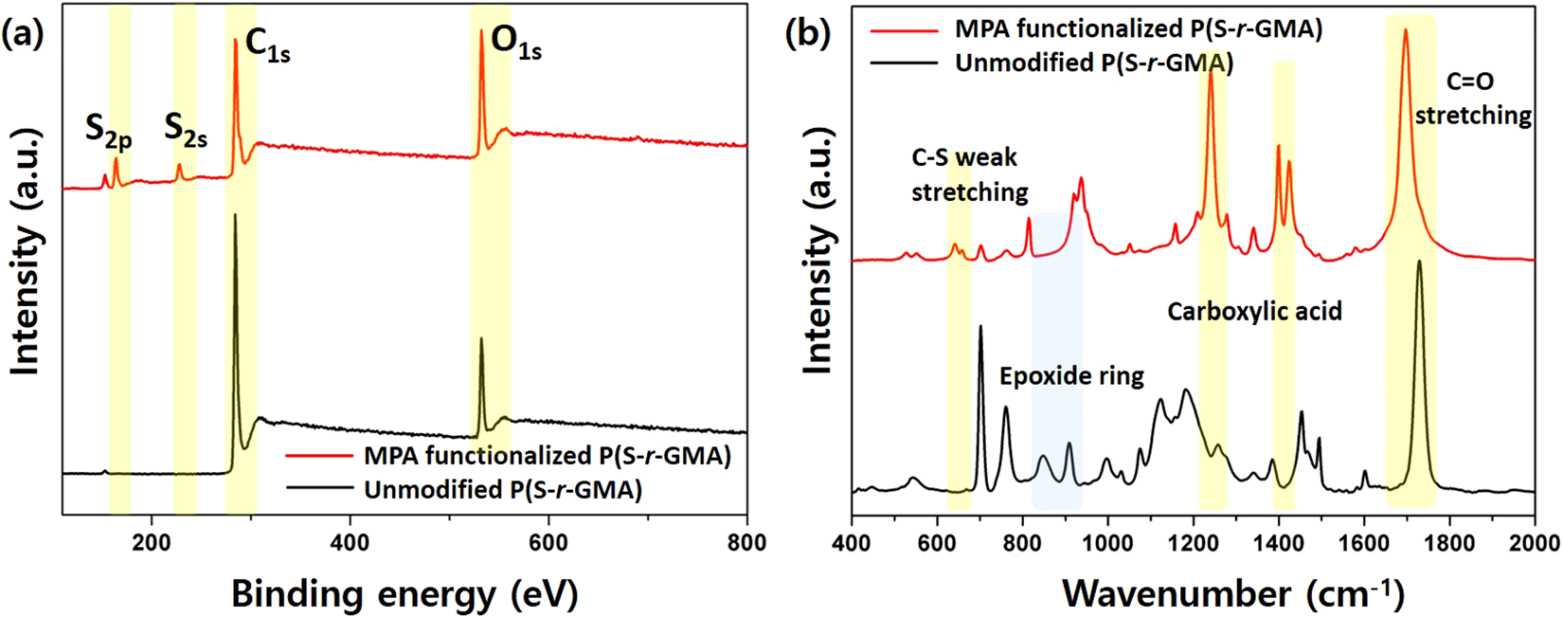
(**a**) XPS survey spectra and (**b**) FT-IR spectra of unmodified P(S-*r*-GMA) and MPA-functionalized P(S-*r*-GMA) particles.

**Figure 8:**
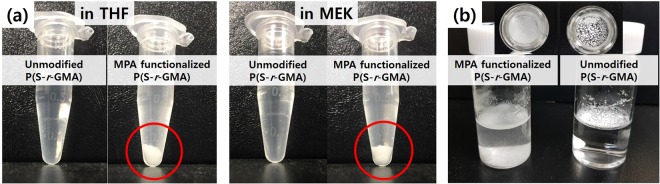
(**a**) Photographic images of unmodified and MPA-functionalized P(S-*r*-GMA) particles treated with (left) THF and (right) methyl ethyl ketone. (**b**) Photographic images showing dispersibilities of (left) MPA-functionalized P(S-*r*-GMA) and (right) unmodified P(S-*r*-GMA) particles in water.

Khan *et al*. reported that thiol-epoxy click reactions afforded quantitative yields within an hour in the presence of LiOH as the alkaline catalyst that deprotonates the thiol to form the thiolate anion^39^. The thiolate nucleophile attacks the less hindered carbon in the epoxide, resulting in ring opening. In the current studies, the thiol-epoxy click reaction between the epoxides in P(S-*r*-GMA) and MPA was conducted with LiOH as the base, and the resulting particles were characterized by XPS and FT-IR techniques. Figure 7a displays XPS survey spectra of the electrosprayed particles before and after functionalization with MPA. The XPS spectrum of the MPA-modified particle is considerably different to that of the unmodified particle, with new peaks observed at ~164 and ~228 eV in the spectrum of the former, which are assigned to S(2p) and S(2s) peaks, respectively. Upon functionalization, an emergence of both peaks, a dramatic increase of O(1 s) peak, and a deconvolution of C(1 s) multiplex spectrum (Fig. S2) confirm the successful incorporation of MPA through the thiol-epoxy click reaction. FT-IR spectra acquired from the particles before and after functionalization also strongly support their successful modification (Fig. 7b). Peaks at 849 and 908 cm^−1^ are characteristic epoxide bands^40^ that are clearly present in the spectrum of the unmodified P(S-*r*-GMA) particles. These peaks clearly disappear upon functionalization, indicating that the epoxides become fully opened during the reaction. The emergence of new peaks at 640–660, 1240, 1400, and 1420 cm^−1^, assigned to the weak S-C stretching mode^41^, the strong C-O stretching mode of the carboxylic acid, the C=O stretching mode of the carboxylate (COO^−^), and the in-plane C-O-H bending mode of the carboxylic acid, respectively, confirm that the click reaction was successful^42,43^. In addition, it is worth noting that the presence of the carboxylic acid upon the reaction is further confirmed by the shift of the ester C=O peak, initially at 1730 cm^−1^, to 1695 cm^−1^ (assigned to the C=O of the carboxylic acid). The functionalized P(S-*r*-GMA) particles exhibit greatly improved dispersibility in water compared to the unmodified P(S-*r*-GMA) particles that float on water, highlighting the impact of surface functionalization (Fig. 8b). Moreover, the complete disappearance of the vibrational modes of the epoxide ring in the FT-IR spectrum is interpreted in two ways: (i) MPA functionalization of the surfaces of the particles, and (ii) crosslinking reactions within the particles. As shown in Fig. 8a, the functionalized particles are not soluble in MEK or THF, solvents that immediately dissolve the unmodified copolymer particles, indicating that the functionalized particles are effectively stabilized by crosslinking, which is an intrinsic virtue of epoxy-containing polymers that easily undergo crosslinking reactions with catalytic LiOH. This reaction is possibly facilitated by the effective penetration of the hydroxide ions from LiOH, which is a strong base and acts as a catalyst that facilitates epoxy ring opening and subsequent crosslinking, leading to polymeric particles that are stable against organic solvents. These results highlight the versatility of epoxy-containing copolymers that provide simple, rapid, and efficient chemical routes for concurrent functionalization and stabilization. In addition, the epoxy chemistry can be further expanded by utilizing the reactions with different nucleophiles such as primary amines and alcohols, further highlighting the potential of this simple chemistry to chemically tune polymeric materials with complex structures^44^.

## Conclusions

We systematically studied the size and surface-smoothness controlled electrospray fabrication of functionalizable and stabilizable polymeric particles that exhibit unconventional and nonspherical morphologies. We designed and successfully synthesized P(S-*r*-GMA) copolymers with various molecular weights and compositions in order to realize a reactive polymer platform toward functionalizable polymeric structures. Synthesized copolymers were subjected to electrospraying; the surface morphologies and sizes of the microparticles produced were effectively controlled by adjusting the feed composition, solvent, feed rate, and applied voltage. We further demonstrated that the fabricated particles can be functionalized with a widely utilized functional thiol, confirming that the reactive epoxides preserved during particle formation can be effectively used to introduce desired chemical functionality. Concurrently, the copolymer particles were stabilized through effective crosslinking with an alkaline catalyst, further highlighting the impact of epoxy chemistry for the formation of functional structures. Our findings emphasize the importance of copolymer design and understanding the relationships between structure and properties in order to fine-tune structural parameters such as the sizes and morphologies of organic micro/nanosized structures. Moreover, the current studies open up possibilities for achieving a variety of organic functionalizable structures.

## Materials and Methods

### Materials

All chemicals were used as received unless otherwise specified. Styrene and glycidyl methacrylate (GMA) were purchased from TCI Chemicals and Sigma-Aldrich, respectively. Azobisisobutyronitrile (AIBN) was purchased from the Junsei Chemical Co., Ltd., and recrystallized from methanol prior to use. Tetrahydrofuran (THF), toluene, methyl ethyl ketone (MEK), and methanol were purchased from Honeywell Chemicals, Samchun Chemicals, Alfa Aesar, and Daejung Chemicals, respectively. 3-Mercaptopropionic acid (MPA), lithium hydroxide (LiOH), polystyrene (PS, M_n_ = 35 kg/mol), and poly(glycidyl methacrylate) (PGMA, M_n_ = 10–20 kg/mol) were purchased from Sigma-Aldrich.

### Synthesis of P(S-*r*-GMA)

A series of P(S-*r*-GMA) copolymers was synthesized by the conventional free-radical polymerization of styrene and GMA^24^. The fraction of styrene (*f*_st_) in the reaction mixture varied between 0 and 1. The amount of initiator and the polymerization time were varied to produce copolymers with the target molecular weights. In a typical procedure, a mixture of monomers with the desired feed ratio, AIBN (~0.6 mol% based on the monomer mixture), and toluene or MEK (~200 wt% based on the monomer mixture) was completely degassed by three freeze-pump-thaw cycles, after which it was heated at 80 °C (oil bath) for the required time. The resulting polymer solution was then diluted with THF and precipitated in methanol. The polymer was collected as a white powder by filtration and dried under vacuum. The samples were further purified with reprecipitations from methanol twice.

### Electrospray and subsequent functionalization

P(S-*r*-GMA) samples were dissolved in three different solvents (acetone, THF, and MEK) at controlled concentrations in the 1–18 wt% range. The polymer solutions were stirred for at least 3 h at room temperature, after which they were electrosprayed in order to synthesize micro- or nano-objects. The electrospray apparatus was equipped with a high-voltage power supply (Har-100*12, Matsusada Co., Tokyo, Japan) that created electric fields by generating voltages up to 100 kV. The positive electrode of the power supply was connected to a stainless steel syringe needle, and the negative electrode was connected to a metallic collector. The polymer electrospray solution was loaded into the syringe and the flow rate through the needle was controlled precisely with a syringe pump. Typical process parameters for electrospray included a tip-to-collector distance, voltage, and flow rate of 15 cm, 11 kV, and 0.9 mL/h, respectively, at room temperature with 40% relative humidity, unless specified otherwise. Droplets that formed by electrospray were collected on aluminum foil that covered the metallic collector. Post-electrospray functionalization was carried out using a thiol-epoxy click reaction using aqueous MPA. The fabricated P(S-*r*-GMA) particles were immersed in an aqueous solution of MPA and LiOH at room temperature for 3 h. The ratio of epoxy groups to thiols was 1:3, and the amount of LiOH in the reaction mixture was 10 mol% based on thiol. The resulting particles were collected and dried under ambient conditions for 24 h and then further washed with water, recollected, and dried under vacuum.

### Characterization

^1^H-NMR spectra were obtained in CDCl_3_ using a JEOL JNM-ECZ400S spectrometer at 400 MHz with TMS as the internal reference. A pulse delay of 10 s was used during the acquisition of spectra for quantitative analyses. Size-exclusion chromatography (SEC) was performed using a Thermo Scientific UltiMate 3000 chromatograph with THF as the eluent at a flow rate of 1 mL/min at 35 °C. The system was calibrated with nine narrow-dispersity polystyrene standard samples with number-average molecular weights between 1 and 1,400 kg/mol. The surface morphologies of the resulting samples were examined by scanning electron microscopy (SEM, JSM-6010LA, JEOL, Tokyo, Japan) and field-emission scanning electron microscopy (FE-SEM, S-5000, Hitachi, Tokyo, Japan). The average sizes of the particles and nanofibers were determined from the SEM images using image-analysis software (ImageJ, Version 1.49). At least 50 points in a single SEM image were randomly selected in order to determine average values and their standard deviations. Chemical compositions of the fabricated materials were examined by X-ray photoelectron spectroscopy (XPS) using a Thermo Scientific K-Alpha instrument. Silicon wafers were used as substrates for the resulting particles. FT-IR spectra were acquired on a IR Prestige-21 spectrometer (Shimadzu Co., Kyoto, Japan) or a Bruker VERTEX 80V instrument.

## Electronic supplementary material

Supplementary Information
