# Supplementary material for: Readily Functionalizable and Stabilizable Polymeric Particles with Controlled Size and Morphology by Electrospray
Source: Sci Rep. 2018 Oct 24;8:15725. doi: 10.1038/s41598-018-34124-0 (PMC6200772; doi:10.1038/s41598-018-34124-0)
Supplement: Supplementary file 1 — Supplementary Information [file 41598_2018_34124_MOESM1_ESM.docx]

Supporting Information
Readily Functionalizable and Stabilizable Polymeric Particles with Controlled Size and Morphology by Electrospray

Hoik Lee,^1^ Sol An,^2^ Sukjoo Kim,^1^ Bokyoung Jeon,^2^ Myungwoong Kim,^2,*^ and Ick Soo Kim.^1,*^

^1^Nano Fusion Technology Research Group, Division of Frontier Fibers, Institute for Fiber Engineering (IFES), Interdisciplinary Cluster for Cutting Edge Research (ICCER), Shinshu University, Tokida 3-15-1, Ueda, Nagano 386-8567, Japan.

^2^Department of Chemistry and Chemical Engineering, Inha University, Incheon 22212, Korea.

*Corresponding authors: Ick Soo Kim (kim@shinshu-u.ac.jp), Myungwoong Kim (mkim233@inha.ac.kr)

**KEYWORDS** Polymer particles; Electrospray deposition; Reactive polymer; Crosslinkable polymer; Morphology control; Functionalizable surface

**
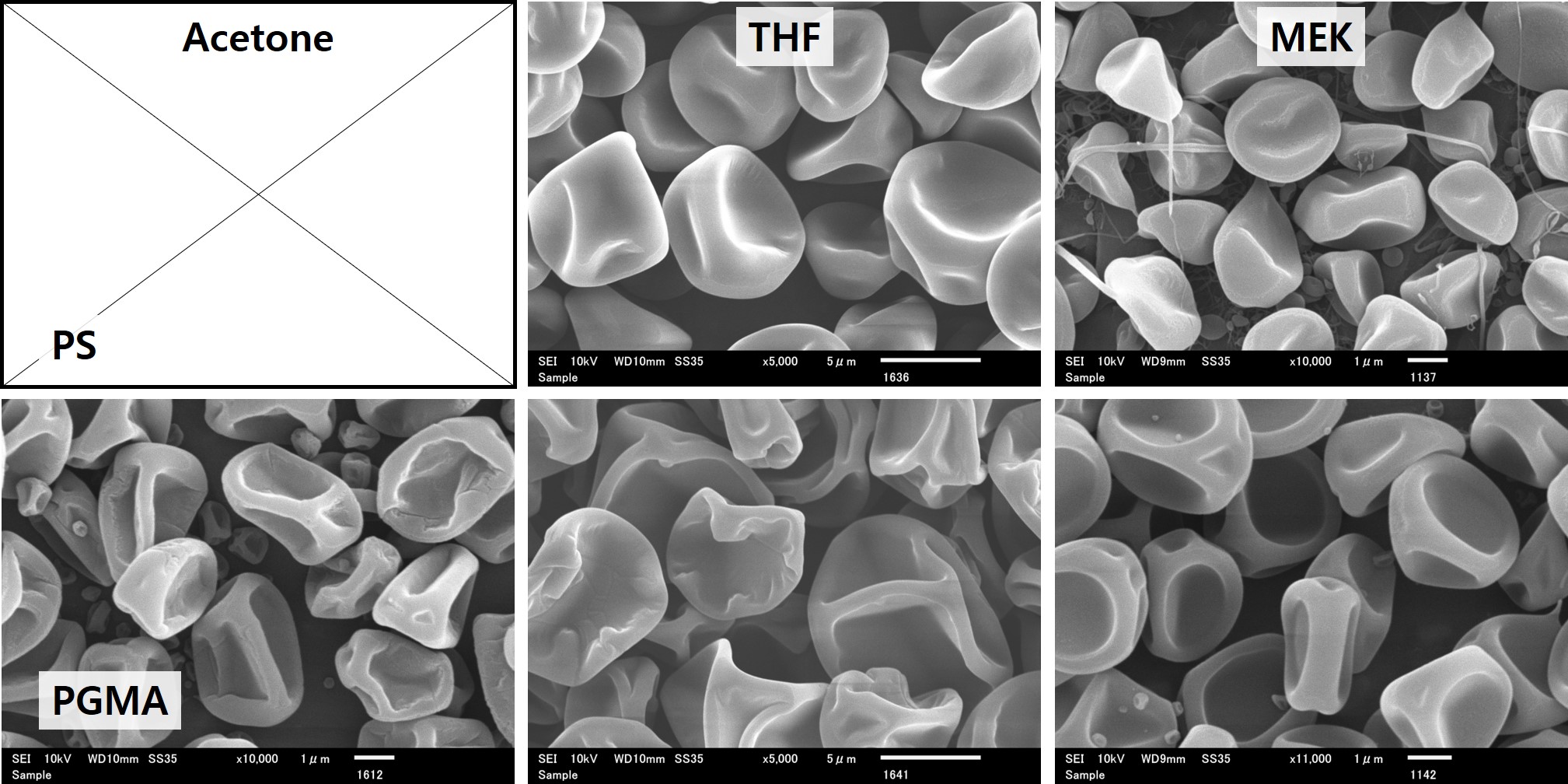
Figure S1**. SEM images of electrosprayed particles: (top) PS in acetone, THF, and MEK, respectively; (bottom) PGMA in acetone, THF, and MEK, respectively.

**
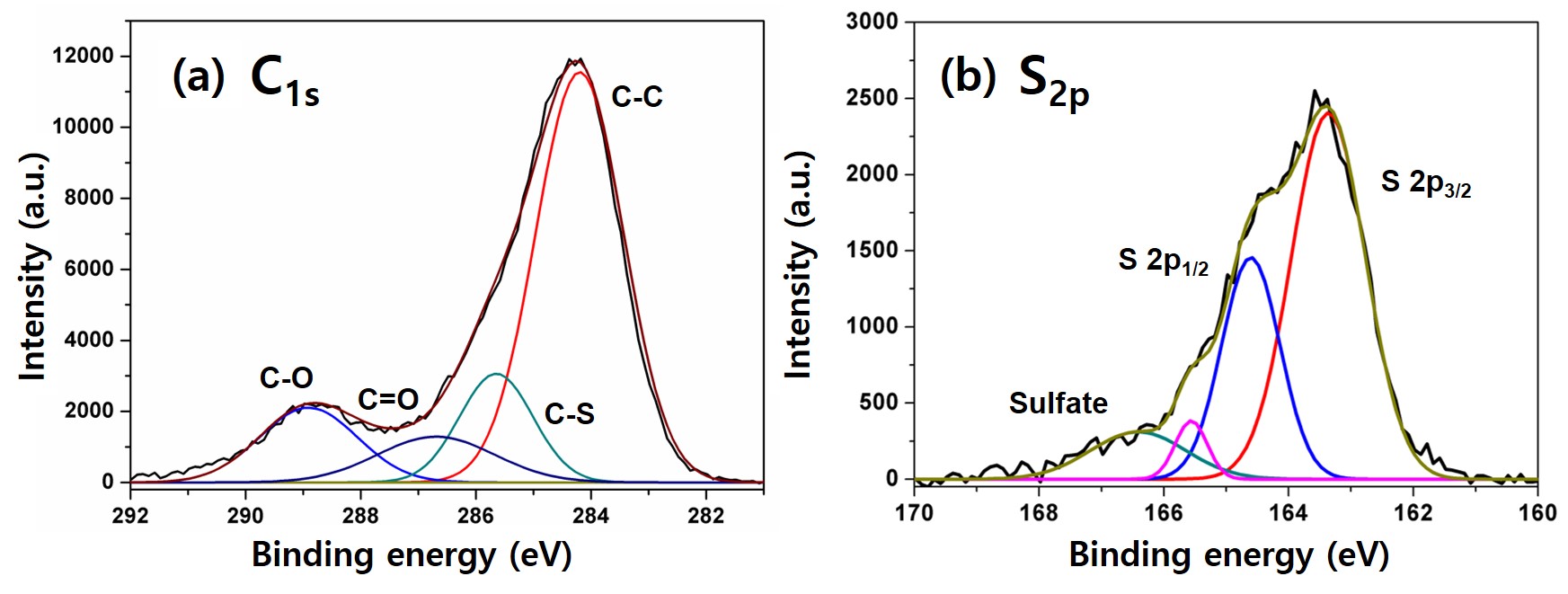
**

**Figure S2**. (a) C(1s) and (b) S(2p) XPS multiplex spectra of MPA-functionalized P(S-*r*-GMA) particles.

**Table S1.** The processing conditions for the experiments presented in from **Figure 2** to **Figure 6**.

|  | Solvent | Concentration(wt%) | Sample | *F_st_* | Tip-to-collector distance (cm) | Voltage (kV) | Feed rate (mL/h) |
| --- | --- | --- | --- | --- | --- | --- | --- |
| Figure 2a | MEK | 1 | SG0.6-60K | 0.6 | 15 | 11 | 0.9 |
| Figure 2b | MEK | 3 | SG0.6-60K | 0.6 | 15 | 11 | 0.9 |
| Figure 2c | MEK | 5 | SG0.6-60K | 0.6 | 15 | 11 | 0.9 |
| Figure 2d | MEK | 10 | SG0.6-60K | 0.6 | 15 | 11 | 0.9 |
| Figure 2e | MEK | 14 | SG0.6-60K | 0.6 | 15 | 11 | 0.9 |
| Figure 2f | MEK | 15 | SG0.6-60K | 0.6 | 15 | 11 | 0.9 |
| Figure 2g | MEK | 16 | SG0.6-60K | 0.6 | 15 | 11 | 0.9 |
| Figure 2h | MEK | 17 | SG0.6-60K | 0.6 | 15 | 11 | 0.9 |
| Figure 2i | MEK | 18 | SG0.6-60K | 0.6 | 15 | 11 | 0.9 |
| Figure 3a | MEK | 5 | SG0.6-15K | 0.6 | 15 | 11 | 0.6 |
| Figure 3b | MEK | 5 | SG0.6-24K | 0.6 | 15 | 11 | 0.6 |
| Figure 3c | MEK | 5 | SG0.6-53K | 0.6 | 15 | 11 | 0.6 |
| Figure 3d | MEK | 5 | SG0.6-126K | 0.6 | 15 | 11 | 0.6 |
| Figure 4a | Acetone | 5 | SG0.6-60K | 0.6 | 15 | 11 | 0.6 |
| Figure 4b | Acetone | 5 | SG0.6-60K | 0.6 | 15 | 11 | 9 |
| Figure 5b | MEK | 5 | SG0.6-60K | 0.6 | 15 | 11 | 0.9 |
| Figure 5b | MEK | 5 | SG0.6-60K | 0.6 | 15 | 13 | 0.9 |
| Figure 5b | MEK | 5 | SG0.6-60K | 0.6 | 15 | 15 | 0.9 |
| Figure 6a | Acetone | 5 | SG0.2-76K | 0.2 | 15 | 11 | 0.9 |
| Figure 6b | THF | 5 | SG0.2-76K | 0.2 | 15 | 11 | 0.9 |
| Figure 6c | MEK | 5 | SG0.2-76K | 0.2 | 15 | 11 | 0.9 |
| Figure 6d | Acetone | 5 | SG0.6-60K | 0.6 | 15 | 11 | 0.9 |
| Figure 6e | THF | 5 | SG0.6-60K | 0.6 | 15 | 11 | 0.9 |
| Figure 6f | MEK | 5 | SG0.6-60K | 0.6 | 15 | 11 | 0.9 |
| Figure 6g | Acetone | 5 | SG0.8-63K | 0.8 | 15 | 11 | 0.9 |
| Figure 6h | THF | 5 | SG0.8-63K | 0.8 | 15 | 11 | 0.9 |
| Figure 6i | MEK | 5 | SG0.8-63K | 0.8 | 15 | 11 | 0.9 |
